# Longitudinal Changes in Serum Procalcitonin After Bariatric Surgery and Their Associations with Anthropometric, Metabolic, and Inflammatory Parameters †

**DOI:** 10.3390/jcm15135293

**Published:** 2026-07-07

**Authors:** Gurbet Ünal Özen, Çağrı Büyükkasap, Beyza Dursun, Aslı Akyol

**Affiliations:** 1 Department of Nutrition and Dietetics, Faculty of Health Sciences, Hacettepe University, Ankara 06100, Turkey; asli.akyol@hacettepe.edu.tr; 2 Department of General Surgery, Faculty of Medicine, Gazi University, Ankara 06560, Turkey; cagribuyukkasap@gazi.edu.tr; 3 Department of Physiology, Faculty of Medicine, Ankara Yildirim Beyazit University, Ankara 06800, Turkey; sbdursun@aybu.edu.tr

**Keywords:** bariatric surgery, sleeve gastrectomy, procalcitonin, inflammation

## Abstract

**Objective**: Obesity is a systemic disease characterized by low-grade chronic inflammation and increased metabolic risk. Although bariatric surgery is known to improve metabolic and inflammatory status, the longitudinal behavior of emerging inflammatory biomarkers such as procalcitonin (PCT) remains insufficiently characterized. This study aimed to evaluate postoperative changes in serum procalcitonin (PCT) levels in patients undergoing bariatric surgery and to investigate their associations with anthropometric measurements, liver enzymes, and novel inflammatory indices. **Methods:** In this retrospective longitudinal cohort study, 38 patients who underwent sleeve gastrectomy and had complete preoperative and postoperative follow-up data at months 1, 3, and 6 were included. Anthropometric and biochemical parameters were analyzed, and systemic inflammation was assessed using PCT, Systemic Immune-Inflammation Index (SII) and the Systemic Inflammation Response Index (SIRI). Repeated-measures analyses were performed according to data distribution, and correlations were evaluated using Spearman analysis. **Results:** PCT levels showed a significant reduction at postoperative month 1 compared with the preoperative period. However, despite continued reductions in body weight, BMI, and fat mass at postoperative months 3 and 6, PCT levels plateaued without further significant change. In the preoperative period, PCT demonstrated strong positive correlations with liver enzymes (*p* < 0.01). At postoperative month 1, PCT was significantly associated with glucose and HbA1c levels. Although SII and SIRI decreased after surgery, no significant correlation with PCT was observed. **Conclusions**: PCT decreased in the early postoperative period after sleeve gastrectomy and may reflect early metabolic and inflammatory changes associated with rapid weight loss. However, its sensitivity for monitoring long-term inflammatory changes appears limited. The observed preoperative associations with liver enzymes may suggest a potential relationship between PCT levels, liver enzyme alterations, and metabolic alterations in obesity.

## 1. Introduction

Obesity is a strong risk factor for insulin resistance, type 2 diabetes mellitus, cardiovascular diseases, immune dysfunction, metabolic dysfunction-associated steatotic liver disease (MASLD), and various malignancies, and it is currently regarded as a global epidemic [1,2]. According to the World Health Organization European Regional Obesity Report 2022, one in every eight adults worldwide is obese, and the prevalence of obesity has more than doubled over the past three decades [3].

Despite lifestyle modifications and pharmacological therapies, the low long-term success rates in sustained weight loss have made bariatric surgery the most effective and durable treatment option, particularly for individuals with a BMI ≥ 40 kg/m^2^ or a BMI ≥ 35 kg/m^2^ with obesity-related comorbidities [4,5]. Procedures such as sleeve gastrectomy and Roux-en-Y gastric bypass not only promote weight loss but are also known to contribute to metabolic improvement and modulation of the inflammatory response. Procedures such as sleeve gastrectomy, Roux-en-Y gastric bypass, and emerging surgical procedures [6], as well as minimally invasive endoscopic and endovascular bariatric procedures, not only promote weight loss but are also known to contribute to metabolic improvement and modulation of the inflammatory response [7].

Procalcitonin (PCT), a 116–amino acid precursor of calcitonin, is synthesized primarily by the parafollicular C cells of the thyroid gland under normal physiological conditions. During systemic inflammatory states, particularly bacterial infections, its production is markedly upregulated in multiple extra-thyroid tissues. PCT is a well-established biomarker of infection and systemic inflammation [8]. Circulating PCT levels are normally very low (≤0.1 ng/mL) but increase markedly during bacterial infections, making PCT a useful marker for infection detection and guidance of antibiotic therapy [8,9,10]. In obesity, the expansion and functional impairment of adipose tissue are key determinants of chronic low-grade systemic inflammation [11,12]. Adipose tissue undergoes a remodeling process characterized by macrophage infiltration, hypoxia, angiogenesis, and increased production of proinflammatory cytokines, which play a critical role in the pathogenesis of metabolic disorders [2]. In this context, adipose tissue is recognized not only as an energy reservoir but also as an active endocrine organ capable of synthesizing various inflammatory biomarkers, including PCT [13,14,15]. Although PCT is normally present at low levels, it is known to increase markedly in response to inflammatory stimuli [15].

In recent years, growing evidence suggests that PCT levels in individuals with obesity are associated with body mass index, fat distribution, and metabolic syndrome parameters [16,17,18]. As adipose tissue mass and inflammatory activity increase, PCT levels also rise; therefore, PCT has been proposed as a potential marker of chronic inflammation in obesity [13,14]. However, the effects of the rapidly changing metabolic and inflammatory environment following bariatric surgery on PCT levels have not been sufficiently investigated.

PCT has been shown to have high sensitivity and specificity in the early detection of surgical complications, such as staple line leakage, in the acute postoperative period following bariatric surgery [19,20,21]. However, data evaluating the temporal changes in PCT levels in the context of chronic low-grade inflammation—beyond acute infection or sepsis—in relation to weight loss and metabolic parameters remain limited. Considering that adipose tissue may contribute to PCT production in obesity, the relationship between postoperative reductions in body composition and changes in circulating PCT levels after bariatric surgery remains unclear. In addition to PCT, the Systemic Immune-Inflammation Index (SII) and the Systemic Inflammation Response Index (SIRI) are included as complementary indicators of systemic inflammation. These indices are derived from routine hematological parameters and have been reported to correlate with established inflammatory markers such as C-reactive protein (CRP) and pro-inflammatory cytokines in various inflammatory and metabolic conditions [22].

Therefore, the aim of this study was to evaluate the changes in serum PCT levels from the preoperative period to postoperative month 6 in patients undergoing bariatric surgery and to investigate their associations with anthropometric measurements, glucose homeostasis, liver enzymes and inflammatory parameters, including the SIRI and SII.

## 2. Materials and Methods

### 2.1. Study Design and Patient Selection

This retrospective longitudinal cohort study was conducted at the Department of General Surgery, Gazi University Hospital, Ankara, Türkiye and included patients who underwent laparoscopic sleeve gastrectomy between 1 January 2022 and 1 January 2024. Only sleeve gastrectomy patients were included to maintain a homogeneous study population and minimize potential variability arising from differences in postoperative metabolic and inflammatory responses associated with different bariatric procedures.

In accordance with the study objective, patients with complete clinical, biochemical, and anthropometric measurements obtained during the routine preoperative evaluation (within one month before surgery) and at postoperative months 1, 3, and 6 were included in the final analysis.

**Figure 1:**
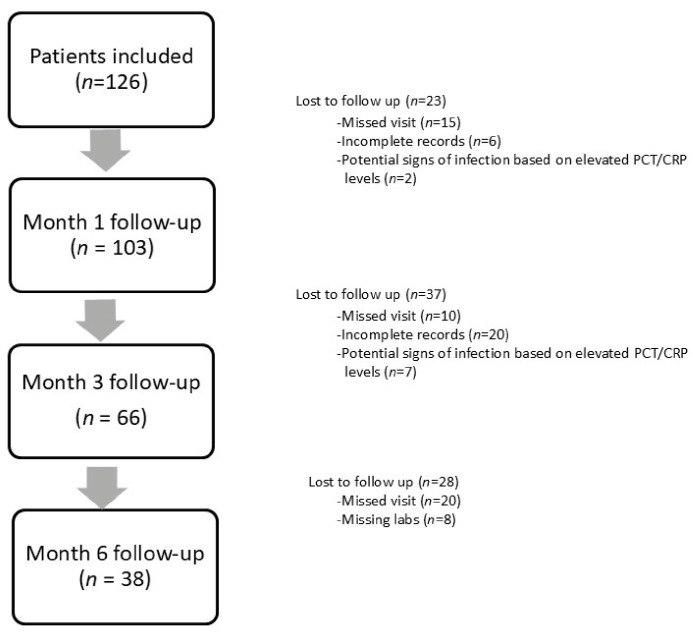
Patient flow diagram.

Patient flow is summarized in Figure 1. Of the 126 patients initially evaluated, 38 completed the 6-month follow-up after excluding those with missing data or not meeting eligibility criteria. Losses during follow-up were due to missed visits, incomplete records, missing laboratory data, and exclusion because of potential signs of infection based on elevated PCT/CRP levels.

To assess the potential risk of selection bias, baseline characteristics of patients included in the final analysis were compared with those of patients excluded because of incomplete follow-up, and the results are presented in Supplementary Table S1.

A total of 126 patients who underwent sleeve gastrectomy were initially included. At postoperative month 1, 103 patients remained available for follow-up after excluding 23 patients because of missed visits (*n* = 15), incomplete records (*n* = 6), or potential signs of infection based on elevated PCT/CRP levels (*n* = 2). At postoperative month 3, 66 patients remained after excluding an additional 37 patients because of missed visits (*n* = 10), incomplete records (*n* = 20), or potential signs of infection based on elevated PCT/CRP levels (*n* = 7). At postoperative month 6, 38 patients with complete follow-up data were included in the final analysis after excluding 28 patients because of missed visits (*n* = 20) or missing laboratory data (*n* = 8). PCT, procalcitonin; CRP, C-reactive protein.

#### 2.1.1. Inclusion Criteria

- Adults aged 18–65 years
- Undergoing sleeve gastrectomy
- Complete laboratory and anthropometric data available for the preoperative period and postoperative months 1, 3, and 6.

#### 2.1.2. Exclusion Criteria

- Presentation with active infection, suspected sepsis, or elevated CRP/PCT levels
- Chronic liver failure, renal failure, thyroid disease, or endocrinopathies that may affect PCT levels
- History of malignancy
- Chronic use of steroids, immunosuppressive, or anti-inflammatory medications
- Development of postoperative complications (e.g., bleeding, leakage, infection)
- Acute illness occurring at measurement time points

### 2.2. Anthropometric Measurements

Body composition analyses were performed using bioelectrical impedance analysis (BIA) with a Tanita BC-780 MA (Tanita Corporation, Tokyo, Japan). body composition analyzer. The measurements included body weight, fat-free mass, muscle mass, fat mass, waist circumference, basal metabolic rate (BMR), and BMI. Assessment of these parameters allowed for a more comprehensive evaluation of postoperative changes in body composition and energy metabolism than could be achieved using body weight and BMI alone.

To ensure measurement accuracy and standardization, the following conditions were observed: patients were measured with minimal clothing; measurements were performed in the fasting state (no food intake for at least 2–4 h); no strenuous physical activity was performed within the preceding 24–48 h; no alcohol consumption occurred within 24 h; tea or coffee was avoided for at least 4 h prior to measurement; no metal objects were worn; and individuals with cardiac pacemakers were excluded [23]. Anthropometric measurement data and biochemical parameters were recorded from patient medical records.

At the time of surgery, 18 patients had prediabetes, 11 had type 2 diabetes mellitus, and 9 had no documented impairment in glucose metabolism. In addition, all patients receiving glucose-lowering medications discontinued these treatments after surgery, and no antidiabetic medications were used during the follow-up period.

### 2.3. Clinical and Laboratory Assessments

Fasting laboratory analyses included PCT, fasting glucose, glycated hemoglobin (HbA1c; IFCC and NGSP), Homeostatic Model Assessment of Insulin Resistance (HOMA-IR), aspartate aminotransferase (AST), alanine aminotransferase (ALT), gamma-glutamyl transferase (GGT), iron, ferritin, platelet count, neutrophil count, lymphocyte count, and monocyte count. These parameters, recorded preoperatively and repeated at postoperative months 1, 3, and 6, were obtained from patient medical records.

CRP measurements were not routinely performed during the study period and were available only for patients evaluated for suspected acute illness or infection. Therefore, CRP data were available for a limited subset of patients and could not be included in the longitudinal analyses.

HOMA-IR was calculated using the formula: fasting insulin (µU/mL) × fasting glucose (mg/dL)/405 [24].

Based on the available data, the SII and the SIRI were also calculated. SII and SIRI were calculated using the formulas platelet count × neutrophil count/lymphocyte count and monocyte count × neutrophil count/lymphocyte count, respectively [25].

### 2.4. Statistical Analysis

Data were analyzed using IBM SPSS Statistics version 22.0 (IBM Corp., Armonk, NY, USA). Descriptive statistics were calculated for all variables. Data distribution characteristics were assessed using the Shapiro–Wilk test.

For repeated measurements with normal distribution, repeated-measures ANOVA was used; variables found to be statistically significant were further evaluated with Bonferroni-adjusted post hoc analyses. For repeated measurements that did not show normal distribution, the Friedman test was applied, and Dunn–Bonferroni post hoc analyses were performed for variables with significant results.

Relationships between variables were analyzed using Spearman’s rank correlation coefficient. Statistical significance was accepted at *p* < 0.05.

The study included 38 patients. A post hoc power analysis was performed based on the primary outcome, defined as the change in serum procalcitonin levels between the preoperative period and postoperative month 1. Based on the observed effect size, the statistical power (1–β) was calculated as 0.96 at a significance level of α = 0.05.

## 3. Results

The study was completed with a total of 38 patients who had complete follow-up data. Of the participants, 84.2% were female and 15.8% were male, with a mean age of 38.34 ± 11.33 years.

### 3.1. Changes in Anthropometric Parameters

To evaluate the effectiveness of bariatric surgery, longitudinal changes in anthropometric parameters were assessed during the 6-month follow-up period. These measures are clinically important indicators of weight-loss success and improvements in obesity-related health outcomes.

Changes in body composition following bariatric surgery are summarized in Table 1. Compared with the preoperative period, body weight, BMI, fat mass, and waist circumference showed a statistically significant and continuous decrease at postoperative months 1, 3, and 6 (*p* < 0.001). Similarly, fat-free mass and BMR demonstrated the expected physiological decline, which was significant across all follow-up periods.

### 3.2. Changes in Inflammatory and Metabolic Parameters over Time

**Figure 2:**
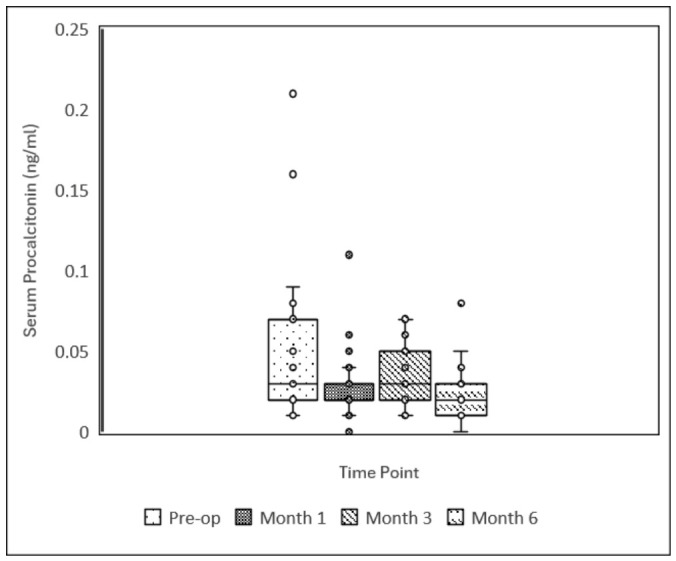
Longitudinal changes in serum procalcitonin levels following bariatric surgery. Serum PCT concentrations were assessed preoperatively and at postoperative months 1, 3, and 6. Boxplots indicate the median and interquartile range, whiskers represent the minimum and maximum values, and open circles denote individual patient values.

To better understand the clinical significance of PCT, correlation analyses were performed between PCT levels and anthropometric, metabolic, hepatic, and inflammatory parameters. Identifying these associations may contribute to the interpretation of PCT beyond its traditional role as a biomarker of infection. Temporal changes in inflammatory markers are presented in Table 2. Median serum PCT levels decreased significantly from 0.037 ng/mL preoperatively to 0.028 ng/mL at postoperative month 1 (*p* < 0.05) (Figure 2).

**Figure 3:**
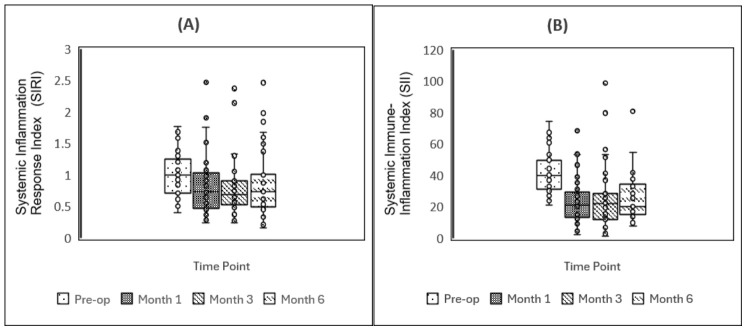
Longitudinal changes in systemic inflammatory indices after bariatric surgery. (**A**) Systemic Inflammation Response Index (SIRI) and (**B**) Systemic Immune-Inflammation Index (SII) measured preoperatively and at postoperative months 1, 3, and 6. Boxplots represent the median and interquartile range, whiskers indicate the minimum and maximum values, and individual circles represent patient-specific measurements.

Despite continued significant weight loss at postoperative months 3 and 6, PCT levels did not decrease further and instead exhibited a plateau pattern after month 3. The change in PCT levels was significant only between the preoperative and postoperative measurements; no statistically significant differences were observed among postoperative months 1, 3, and 6 (Table 2, Figure 3).

Other inflammatory indices, SIRI and SII, followed a similar pattern, showing a significant decrease at postoperative month 1 compared with the preoperative period (*p* < 0.001), with no further statistically significant changes in subsequent months (Table 2).

Ferritin levels showed a significant increase at postoperative months 1 and 3 compared with preoperative values, while values approached baseline levels by month 6 (*p* = 0.013) (Table 2).

Parameters related to glucose metabolism (fasting glucose, HbA1c, HOMA-IR, and insulin) demonstrated significant postoperative reductions (*p* < 0.001) (Table 2). Fasting plasma glucose levels decreased progressively after surgery and were significantly lower at postoperative months 3 and 6 compared with preoperative values (*p* < 0.001). HbA1c (IFCC) values showed a significant decline beginning at postoperative month 1 (*p* < 0.001), and this reduction persisted throughout the follow-up period. Similarly, HbA1c (%) values decreased significantly from a median of 5.9 preoperatively to 5.7, 5.44, and 5.6 at postoperative months 1, 3, and 6, respectively (*p* < 0.001). HOMA-IR values, an indicator of insulin resistance, also showed a marked and statistically significant decrease postoperatively (*p* < 0.001). Serum insulin levels declined significantly after surgery, with the lowest values observed at postoperative month 6 (*p* < 0.001).

Evaluation of liver enzymes revealed significant time-dependent changes postoperatively. ALT and GGT levels demonstrated a significant decrease, particularly at postoperative month 6 (*p* < 0.001) (Table 2).

### 3.3. Correlation Analyses

To better understand the clinical significance of PCT, correlation analyses were performed between PCT levels and anthropometric, metabolic, hepatic, and inflammatory parameters. Identifying these associations may contribute to the interpretation of PCT beyond its traditional role as a biomarker of infection.

Correlation results between PCT and SII and SIRI are presented in Table 3. No significant correlations were found between PCT and SII at any time point. Similarly, no significant relationship was observed between PCT and SIRI.

The relationship between PCT levels and biochemical parameters is detailed in Table 4. In the preoperative period, strong positive correlations were found between PCT and liver enzymes (AST: *r* = 0.494, *p* = 0.002; ALT: *r* = 0.613, *p* < 0.001; GGT: *r* = 0.617, *p* < 0.001). Additionally, PCT showed a significant positive correlation with ferritin (*r* = 0.542, *p* < 0.001) and a significant negative correlation with iron (*r* = −0.416). At postoperative month 1, PCT demonstrated significant positive correlations with glucose (*r* = 0.462, *p* = 0.003) and HbA1c-IFCC (*r* = 0.618, *p* < 0.001).

## 4. Discussion

Obesity is a systemic disease characterized by dysfunctional expansion of adipose tissue accompanied by chronic low-grade inflammation [26]. The demonstration that adipose tissue has the capacity to synthesize and secrete PCT [16,17,18] has raised interest in the role of this molecule in the metabolic recovery process following obesity surgery. In this study, the dynamic relationship between postoperative PCT levels and anthropometric measurements, liver function tests, SII, SIRI, and glucose metabolism was investigated.

One of the significant findings of the present study was the significant reduction in PCT levels, SII/SIRI scores, and body weight during the first postoperative month (Table 1 and Table 2). The decrease observed in the first postoperative month may reflect the attenuation of obesity-related inflammatory burden. These results may suggest that PCT levels are associated with certain metabolic and inflammatory changes in obesity; however, further studies are needed to clarify their potential clinical relevance.

However, although the decrease in PCT was statistically significant, the absolute magnitude of change was modest (0.037 to 0.028 ng/mL) and remained well below the thresholds commonly used for bacterial infection or sepsis [27]. Therefore, these findings should not be interpreted as having direct clinical implications for infection diagnosis. Instead, they may reflect subtle metabolic and inflammatory adaptations occurring during the early postoperative period.

Previous studies have reported a positive association between the severity of obesity and circulating PCT concentrations [16,18]. However, this relationship was not confirmed in the present study. Correlations between BMI and PCT levels were weak and statistically non-significant at all evaluated time points (Supplementary Figure S1). These findings suggest that factors other than BMI may contribute to the observed changes in circulating PCT levels following bariatric surgery.

Nevertheless, the significant early postoperative reductions observed in PCT, SII, and SIRI may still suggest that PCT reflects certain obesity-related metabolic and inflammatory changes. Given the exploratory nature of the present study, the underlying mechanisms and potential clinical significance of these findings require further investigation.

Furthermore, earlier studies have demonstrated that obesity is positively associated with systemic inflammatory indices such as SII and SIRI [28,29]. In accordance with these reports, the decreases observed in SII and SIRI scores in our cohort support the hypothesis that obesity-related systemic inflammation is attenuated in the early postoperative period following sleeve gastrectomy.

However, a different pattern was observed at months 3 and 6. Despite the continued reductions in body weight, BMI, and fat mass (Table 1), the trajectory of PCT levels did not parallel these anthropometric improvements. To better understand this plateau phenomenon, several potential mechanisms can be considered. The initial postoperative period may be accompanied by reductions in the obesity-related inflammatory burden, which could contribute to the decrease in PCT levels [17]. In later phases, as adipose tissue inflammation gradually normalizes and non-adipose sources become relatively more prominent, further improvements in anthropometric parameters may exert little additional effect on PCT concentrations. Consequently, PCT levels may reach a plateau near the assay’s lower detection range, consistent with the low baseline values reported in non-acutely ill populations [16,30]. Another observed finding of the present study was the strong preoperative association observed between PCT levels and liver enzymes (Table 4). The observed preoperative correlations between PCT and liver enzymes may reflect an association between PCT levels and liver enzyme alterations or broader metabolic processes related to obesity. These findings are consistent with previous studies emphasizing the relationship between systemic inflammation and liver injury [31] as well as evidence indicating that GGT levels increase in parallel with inflammatory burden [32]. Although some individuals exhibited elevated transaminase or GGT levels, median preoperative AST (20 U/L), ALT (19 U/L), and GGT (26.5 U/L) values were generally within conventional reference ranges [33]. Therefore, the observed associations between PCT and liver enzymes may reflect subclinical obesity-related hepatic metabolic alterations rather than overt liver injury.

Interestingly, this correlation disappeared in the postoperative period as liver enzyme levels returned toward normal ranges. This observation may indicate that postoperative improvement in liver enzyme levels is accompanied by changes in PCT levels; however, the clinical significance of this relationship requires further investigation.

In our study, no significant associations were observed between serum PCT levels and total cholesterol, LDL cholesterol, HDL cholesterol, non-HDL cholesterol, VLDL cholesterol, or triglyceride levels at any time point. Although dyslipidemia is recognized as an important component of obesity and cardiometabolic risk, our findings suggest that PCT may be associated with metabolic processes other than changes in lipid metabolism. Therefore, the utility of PCT as a marker of lipid metabolism in patients undergoing bariatric surgery may be limited. Nevertheless, lipid metabolism remains a major determinant of long-term cardiovascular risk in individuals with obesity. Recent evidence has demonstrated that achieving lower LDL-C levels is associated with substantial cardiovascular benefits, whereas inappropriate discontinuation or de-escalation of lipid-lowering therapy may expose patients to increased residual cardiovascular risk [34]. Furthermore, although bariatric surgery substantially reduces cardiometabolic and cardiovascular risk, a modest increase in cardiovascular risk over time has been reported during long-term follow-up, suggesting that residual cardiovascular risk may not be completely eliminated despite significant metabolic improvement [35].

In the present study, none of the patients received statins, ezetimibe, PCSK9 inhibitors, or other lipid-lowering agents during the postoperative follow-up period; therefore, the observed changes in PCT levels are unlikely to have been influenced by such therapies. Future studies should further investigate the interplay between inflammatory biomarkers, lipid metabolism, and cardiovascular risk following bariatric surgery.

At postoperative month 1, a positive association between PCT levels and glucose as well as HbA1c (%) was observed. A previous study reported elevated PCT levels in patients with diabetes [36], and glycemic control is known to be closely linked to inflammatory processes [37]. In the present study, 18 patients had prediabetes and 11 had type 2 diabetes mellitus at the time of surgery. However, all glucose-lowering medications were discontinued after surgery, and no patient received antidiabetic treatment during the follow-up period. Bariatric and metabolic surgery enables early reduction or discontinuation of medications used for many obesity-related comorbidities [38]. Therefore, medication-related confounding is unlikely to fully explain the correlations observed between PCT and glucose as well as HbA1c at postoperative month 1. These transient associations may reflect the early metabolic adaptations that occur following sleeve gastrectomy. Bariatric surgery has been shown to improve insulin resistance, insulin secretion, and glucose metabolism through both weight loss-dependent and weight loss-independent mechanisms [39]. Such metabolic changes may have contributed to the temporary associations observed between PCT and glycemic parameters during the early postoperative period. On the other hand, the absence of similar correlations at later follow-up time points suggests that this relationship may be limited to the early postoperative phase. Accordingly, these observations should be interpreted with caution, as their clinical significance remains uncertain.

Ferritin, another marker of inflammation, demonstrated a positive correlation with PCT in the preoperative period (Table 4). This finding further supports the well-known role of ferritin as an acute-phase reactant [40]. Conversely, the negative association observed between serum iron and PCT in the preoperative period may reflect the suppressive effect of obesity-related inflammation on iron metabolism. In obesity, adipose tissue-derived cytokines, particularly IL-6, contribute to increased hepcidin production, thereby promoting functional iron deficiency and disturbances in iron homeostasis [41]. As a result, circulating iron levels decrease while ferritin levels remain elevated. This mechanism explains the pathophysiological process commonly referred to as anemia of chronic disease [42].

Moreover, the systemic inflammatory indices SII and SIRI showed a significant decrease in the first postoperative month (Table 2), but did not demonstrate a significant correlation with PCT levels (Table 3). Nevertheless, both parameters exhibited a similar downward trend during the postoperative follow-up period. This observation suggests that although PCT, SII, and SIRI reflect different biological mechanisms, they may collectively indicate a reduction in the systemic inflammatory response following sleeve gastrectomy. Therefore, even though a direct correlation between these markers was not identified, both may reflect different aspects of the decline in inflammatory burden during the postoperative period.

Beyond its potential association with obesity-related metabolic and inflammatory changes, PCT may also have broader clinical relevance in contemporary bariatric surgery practice. Previous studies have demonstrated that PCT may be useful for the early detection of postoperative complications following bariatric surgery, particularly staple-line and anastomotic leaks. In a prospective study of patients undergoing laparoscopic sleeve gastrectomy, PCT showed higher diagnostic accuracy than conventional inflammatory markers such as CRP, fibrinogen, and white blood cell (WBC) count for the early detection of gastric leaks [21]. Similarly, another study reported that PCT is a highly specific biomarker for the early identification of postoperative complications and may provide complementary diagnostic value when interpreted alongside conventional markers such as CRP, WBC, and ΔWBC [43]. Furthermore, Enhanced Recovery After Surgery (ERAS) protocols, which aim to reduce surgical stress, accelerate recovery, and optimize perioperative outcomes through evidence-based multidisciplinary care pathways [44], increasingly favor drain-free bariatric surgery strategies. These approaches rely on structured clinical surveillance, biomarker monitoring, and selective imaging rather than routine drain placement for early complication detection [45]. Within this context, biomarkers such as PCT may contribute to postoperative risk stratification and support timely clinical decision-making [43,45]. Although postoperative complications were excluded from the present study, our findings suggest that PCT may be relevant not only as a marker of metabolic and inflammatory alterations following sleeve gastrectomy but also as a potentially useful component of perioperative monitoring strategies in bariatric surgery.

The strengths of the present study include the longitudinal evaluation of patients at multiple time points and the comprehensive analysis of anthropometric, biochemical, and inflammatory parameters. However, several limitations should be acknowledged.

Furthermore, although statistically significant, the observed reduction in PCT was relatively small, and its clinical significance in the context of obesity-related low-grade inflammation remains uncertain.

Body composition was assessed using bioelectrical impedance analysis (BIA), which has known limitations in individuals with obesity due to potential inaccuracies related to altered hydration status and body composition [46]. To minimize variability, all measurements were performed under standardized conditions, including a fasting state and avoidance of recent physical activity. Despite these limitations, BIA remains a practical and widely used method; however, its findings should be interpreted with caution [47].

The relatively small sample size and the predominance of female participants (84.2%) may limit the generalizability of the findings. Sex-related differences in body composition, hormonal regulation, and inflammatory responses may influence PCT levels and metabolic outcomes. Therefore, the results may not be fully generalizable to male patients, and further studies with more balanced sex distributions are warranted. Nevertheless, to address this limitation, an additional subgroup analysis including only female patients was performed, and the longitudinal changes observed in this subgroup were generally consistent with those obtained in the overall cohort (Supplementary Table S2, Figure S2).

Furthermore, although PCT is known to have high specificity for bacterial infections, it may not adequately reflect the subclinical inflammation associated with obesity. The inability to simultaneously measure other inflammatory biomarkers, such as C-reactive protein (CRP), interleukin-6 (IL-6), and tumor necrosis factor-α (TNF-α), represents another limitation of the study.

Finally, given the number of correlation analyses performed, the possibility of type I error cannot be excluded, and these findings should be interpreted with caution. Overall, the correlation analyses performed in the present study should be considered exploratory and hypothesis-generating rather than confirmatory.

## 5. Conclusions

This study demonstrated that PCT levels decreased significantly during the early period following sleeve gastrectomy, although this decrease did not follow a linear pattern. PCT may reflect early postoperative changes associated with weight loss and attenuation of obesity-related inflammatory burden, particularly within the first postoperative month. However, the relatively stable PCT levels observed from the third month onward suggest that its usefulness for monitoring longer-term inflammatory changes remains uncertain. The preoperative correlations observed between PCT and liver enzymes may indicate an association between PCT levels and obesity-related metabolic alterations; however, these findings should be interpreted with caution and do not imply causality. In addition, the association between PCT and glucose as well as HbA1c levels at postoperative month 1 suggests that PCT may be linked to early metabolic and inflammatory changes following bariatric surgery. Overall, although PCT may reflect early postoperative biological changes, its role as a routine biomarker of obesity-related inflammation remains uncertain and requires further investigation. Importantly, the present findings should be interpreted within the context of sleeve gastrectomy and should not be extrapolated to other bariatric procedures, such as Roux-en-Y gastric bypass, without further evidence. Larger prospective studies involving different bariatric techniques are needed to clarify the potential clinical relevance and generalizability of these findings.

## Figures and Tables

**Table 1:** Changes in Anthropometric and Body Composition Parameters After Bariatric Surgery.

Parameters (Mean ± SD)	Preoperative	Postoperative Month 1	Postoperative Month 3	Postoperative Month 6	*p* Value
Body Weight (kg)	109.05 ± 17.45 ^a^	98.21 ± 16.71 ^b^	88.58 ± 15.93 ^c^	80.74 ± 16.54 ^d^	<0.001
BMI (kg/m^2^)	40.95 ± 5.83 ^a^	36.89 ± 5.59 ^b^	33.28 ± 5.49 ^c^	30.36 ± 5.97 ^d^	<0.001
Waist Circumference (cm)	124.26 ± 17.62 ^a^	114.68 ± 16.38 ^b^	103.24 ± 15.45 ^c^	94.63 ± 16.63 ^d^	<0.001
Fat Mass (kg)	46.66 ± 11.82 ^a^	40.32 ± 11.16 ^b^	32.72 ± 10.48 ^c^	26.99 ± 11.56 ^d^	<0.001
Fat-Free Mass (kg)	62.29 ± 8.66 ^a^	57.91 ± 8.56 ^b^	55.86 ± 8.27 ^c^	53.74 ± 7.98 ^d^	<0.001
Muscle Mass (kg)	59.18 ± 8.26 ^a^	54.99 ± 8.16 ^b^	53.06 ± 7.87 ^c^	51.02 ± 7.60 ^d^	<0.001
BMR (kcal)	1942 ± 281 ^a^	1794 ± 271 ^b^	1708 ± 258 ^c^	1627 ± 249 ^d^	<0.001

Data are presented as mean ± standard deviation (mean ± SD). Changes over time were analyzed using one-way repeated measures ANOVA. When a significant overall effect was detected, Bonferroni-adjusted post hoc comparisons were performed. Different superscript letters (a, b, c, d) within the same row indicate statistically significant differences between time points (*p* < 0.05), whereas values sharing at least one superscript letter are not significantly different. BMI: Body Mass Index; BMR: Basal Metabolic Rate.

**Table 2:** Changes in Inflammatory and Metabolic Parameters Over Time.

Parameters	Preoperative	Postoperative Month 1	Posteropative Month 3	Postoperative Month 6	*p* Value
Inflammatory Markers	Median (Min–Max)	Median (Min–Max)	Median (Min–Max)	Median (Min–Max)	
Procalcitonin (ng/mL)	0.037 ^a^ (0.01–0.21)	0.028 ^b^ (0.00–0.11)	0.033 ^b^ (0.00–0.16)	0.033 ^b^ (0.00–0.30)	0.036
SII Index	39.58 ^a^ (21.15–74.43)	20.37 ^b^ (2.15–68.18)	21.16 ^b^ (1.29–98.61)	19.84 ^b^ (7.76–80.84)	<0.001
SIRI Index	1.000 ^a^ (0.40–1.77)	0.730 ^b^ (0.24–2.47)	0.695 ^b^ (0.24–2.37)	0.730 ^b^ (0.16–3.46)	<0.001
Ferritin (ng/mL)	23.25 ^a^ (0.85–142.00)	41.35 ^b^ (6.10–131.00)	38.40 ^b^ (3.12–210.00)	33.99 ^ab^ (0.44–171.60)	0.013
Iron	52 ^a^ (4.35–121.3)	70.2 ^b^ (29–124)	76 ^b^ (44–126)	79 ^b^ (24–161)	<0.001
Glucose Metabolism					
Glucose (mg/dL)	93.00 ^a^ (69.00–289.00)	91.00 ^a^ (62.00–128.00)	85.59 ^b^ (59.00–122.00)	83.50 ^b^ (69.00–94.00)	<0.001
HbA1c (IFCC, mmol/mol)	41.00 ^a^ (13.11–116.40)	38.82 ^b^ (31.20–53.00)	35.98 ^b^ (31.20–41.10)	37.70 ^b^ (29.43–53.00)	<0.001
HbA1c (%)	5.9 ^a^ (2.63–12.8)	5.7 ^b^ (5.01–7.0)	5.44 ^b^ (5.01–5.91)	5.6 ^b^ (4.84–7.0)	<0.001
HOMA-IR	4.27 ^a^ (0.78–40.13)	2.77 ^b^ (0.22–18.79)	2.77 ^b^ (0.22–18.79)	1.78 ^b^ (0.19–5.38)	<0.001
Insulin (µIU/mL)	19.20 ^a^ (3.35–147.75)	12.49 ^ab^ (1.03–565.00)	10.24 ^b^ (4.18–21.73)	9.13 ^b^ (0.94–24.47)	<0.001
C peptide	3.77 ^a^ (1.33–15.46)	2.96 ^a^ (1.19–8.1)	2.72 ^b^ (1.61–5.4)	2.5 ^b^ (1.1–5.34)	<0.001
Lipid Profile					
Triglycerides (mg/dL)	137 ^a^ (46–517)	114 ^ab^ (71–278)	116 ^b^ (46.1–239)	102 ^b^ (65–254)	<0.001
LDL cholesterol (mg/dL)	111.0 ^ab^ (2–192)	105.43 ^b^ (62–237)	130.0 ^a^ (51–205)	116.0 ^ab^ (77–193)	0.009
HDL cholesterol (mg/dL)	45.50 ^a^ (32–69)	38.6 ^b^ (26.9–59)	42.1 ^ab^ (28.0–61.2)	48.9 ^a^ (29.8–53)	
VLDL cholesterol (mg/dL)	27.00 ^a^ (9–103)	22 ^b^ (14–56)	23 ^ab^ (12–48)	22 ^b^ (16–65)	0.002
Non-HDL cholesterol (mg/dL)	142.0 ^a^ (42–271)	130 ^b^ (19–275)	149.0 ^a^ (89–247)	138 ^ab^ (15–244)	0.008
Total cholesterol (mg/dL)	181.50 ^a^ (95–323)	171.00 ^b^ (116–334)	203.00 ^a^ (119–285)	181.00 ^ab^ (139–291)	0.001
Liver Enzymes					
AST (U/L)	20.00 ^ab^ (11.00–134.00)	24.50 ^a^ (11.55–72.00)	20.00 ^b^ (13.00–36.00)	19.00 ^b^ (11.00–31.00)	<0.001
ALT (U/L)	19.00 ^a^ (10.00–122.00)	31.00 ^b^ (9.63–121.00)	25.01 ^b^ (8.00–57.00)	19.00 ^a^ (2.20–132.00)	<0.001
GGT (U/L)	26.50 ^a^ (5.00–82.00)	20.50 ^a^ (3.00–66.00)	16.00 ^a^ (1.71–94.00)	13.37 ^b^ (2.31–36.70)	<0.001

Data are presented as median (minimum–maximum). Changes over time were analyzed using the Friedman test. When a significant overall effect was detected, pairwise comparisons were performed using the Dunn–Bonferroni post hoc test. Different superscript letters (a, b) within the same row indicate statistically significant differences between time points (*p* < 0.05), whereas values sharing at least one superscript letter are not significantly different. SII: Systemic Immune-Inflammation Index; SIRI: Systemic Inflammation Response Index.

**Table 3:** Relationships Between Procalcitonin and SIRI/SII Indices.

	Preoperative		Postoperative Month 1		Postoperative Month 3		Postoperative Month 6	
	*r*	*p*	*r*	*p*	*r*	*p*	*r*	*p*
SIRI	0.036	0.832	0.086	0.610	−0.175	0.293	0.109	0.514
SII	0.163	0.330	0.189	0.256	0.012	0.941	−0.217	0.191

The relationships between procalcitonin levels and the SIRI and SII indices were evaluated at each time point using Spearman’s correlation analysis. Correlation coefficients (*r*) and corresponding *p* values are presented. A *p* value < 0.05 was considered statistically significant. SIRI: Systemic Inflammation Response Index; SII: Systemic Immune-Inflammation Index.

**Table 4:** Correlations Between Procalcitonin and Biochemical Parameters.

	Preop		Post-op Month 1		Post-op Month 3		Post-op Month 6	
	*r*	*p*	*r*	*p*	*r*	*p*	*r*	*p*
**Glucose** **(mg/dL)**	−0.032	0.847	0.462 *	0.003	−0.024	0.887	0.115	0.491
AST (U/L)	0.494 *	0.002	0.222	0.180	0.165	0.321	0.221	0.182
ALT (U/L)	0.613 *	<0.001	0.100	0.552	0.129	0.441	0.244	0.140
GGT (U/L)	0.617 *	<0.001	0.203	0.221	0.016	0.923	0.111	0.507
Iron (µg/dL)	−0.416 *	0.009	0.111	0.507	0.164	0.327	−0.234	0.157
Ferritin (ng/mL)	0.542 *	<0.001	−0.252	0.127	−0.093	0.579	0.003	0.985
C-peptide (ng/mL)	0.443 *	0.005	0.318	0.051	−0.027	0.871	0.158	0.344
HbA1c (NGSP, %)	0.328 *	0.044	0.263	0.110	0.127	0.449	0.200	0.228
HbA1c (IFCC, mmol/mol)	0.072	0.669	0.618 *	<0.001	0.140	0.402	0.283	0.085
Total cholesterol (mg/dL)	−0.099	0.554	0.157	0.347	0.027	0.874	−0.048	0.776
Triglycerides (mg/dL)	0.288	0.079	0.279	0.090	0.142	0.395	0.140	0.401
LDL cholesterol (mg/dL)	0.019	0.908	0.171	0.305	0.301	0.066	0.059	0.726
HDL cholesterol (mg/dL)	−0.287	0.081	0.034	0.842	−0.072	0.669	−0.045	0.787
VLDL cholesterol (mg/dL)	0.270	0.101	0.236	0.155	0.155	0.354	0.168	0.315
Non-HDL cholesterol (mg/dL)	0.083	0.620	0.161	0.336	0.244	0.141	0.008	0.962

Correlations between procalcitonin levels and biochemical parameters were assessed at each time point using Spearman’s correlation analysis. Correlation coefficients (*r*) and corresponding *p* values are shown. An asterisk (*) indicates statistical significance (*p* < 0.05). Positive *r* values indicate direct correlations. whereas negative *r* values indicate inverse correlations.

## Data Availability

The datasets generated and/or analyzed during the current study are available from the corresponding author on reasonable request. Data are not publicly available due to institutional and ethical regulations.

## Supplementary Materials

The following supporting information can be downloaded at: https://www.mdpi.com/article/10.3390/jcm15135293/s1, Table S1. Comparison of baseline characteristics between included and excluded patients; Table S2. Longitudinal changes in anthropometric, inflammatory, metabolic, lipid, and liver-related parameters in female patients (*n* = 33) undergoing sleeve gastrectomy; Figure S1. Relationship between serum procalcitonin concentrations and body mass index (BMI) at the preoperative period and postoperative months 1, 3, and 6; Figure S2. Longitudinal Changes in PCT Levels in Female Patients.

## Abbreviations

The following abbreviations are used in this manuscript:

ALT	Alanine aminotransferase
AST	Aspartate aminotransferase
BIA	Bioelectrical impedance analysis
BMI	Body mass index
BMR	Basal metabolic rate
CRP	C-reactive protein
ERAS	Enhanced Recovery After Surgery
GGT	Gamma-glutamyl transferase
HbA1c	Glycated hemoglobin
HOMA-IR	Homeostatic Model Assessment of Insulin Resistance
IFCC	International Federation of Clinical Chemistry and Laboratory Medicine
NGSP	National Glycohemoglobin Standardization Program
PCT	Procalcitonin
SII	Systemic Immune-Inflammation Index
SIRI	Systemic Inflammation Response Index
