# Supplementary material for: Longitudinal Changes in Serum Procalcitonin After Bariatric Surgery and Their Associations with Anthropometric, Metabolic, and Inflammatory Parameters
Source: J Clin Med. 2026 Jul 7;15(13):5293. doi: 10.3390/jcm15135293 (PMC13362668; doi:10.3390/jcm15135293)

Supplementary Material

Table S1. Comparison of baseline characteristics between included and excluded patients.

**Variable**	**Included patients (n = 38)**	**Excluded patients (n = 88)**	**p-value**
Age (years)	37.5 ± 9.2	39.1 ± 10.1	0.412
Female sex, n (%)	32 (84.2)	71 (80.7)	0.645
BMI (kg/m^2)	40.95 ± 5.83	43.5 ± 6.1	0.563
Type 2 diabetes mellitus, n (%)	11 (28.9)	24 (27.3)	0.854
Prediabetes, n (%)	18 (47.4)	39 (44.3)	0.751
Fasting glucose (mg/dL)	93 (69–289)	96 (72–265)	0.529
HbA1c-IFCC (mmol/mol)	41.0 (13.1–116.4)	39.8 (15.2–108.5)	0.611
AST (U/L)	20 (11–134)	22 (10–126)	0.482
ALT (U/L)	19 (10–122)	21 (8–131)	0.536
GGT (U/L)	26.5 (5–82)	28.0 (4–89)	0.448
Serum PCT (ng/mL)	0.037 (0.020–0.110)	0.039 (0.018–0.105)	0.587

Figure S1. Relationship between serum procalcitonin concentrations and body mass index (BMI) at the preoperative period and postoperative months 1, 3, and 6.

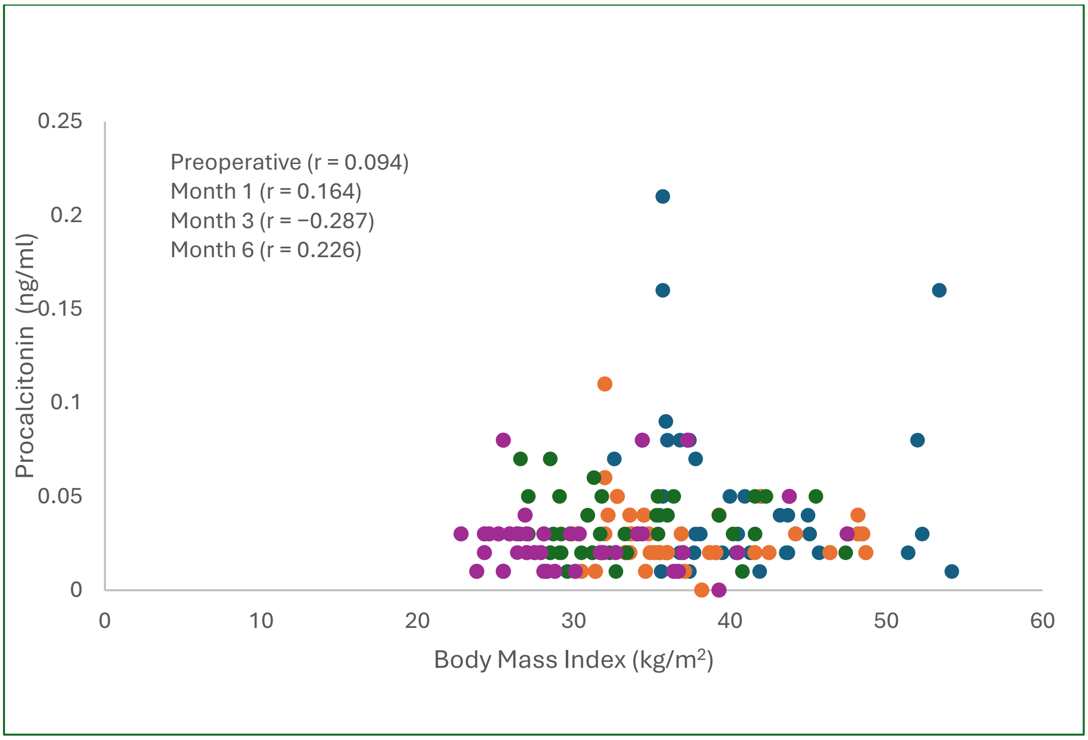

Table S2. Longitudinal changes in anthropometric, inflammatory, metabolic, lipid, and liver-related parameters in female patients (n = 33) undergoing sleeve gastrectomy

Parameter	Preoperative	Postoperative Month 1	Postoperative Month 3	Postoperative Month 6	p value
**Anthropometric Parameters**					
Body weight (kg)	110.0[a] ± 16.9	99.2[b] ± 15.7	89.2[c] ± 14.6	80.9[d] ± 14.8	<0.001
BMI (kg/m²)	40.9[a] ± 5.8	36.92[b] ± 5.4	33.1[c] ± 5.1	30.1[d] ± 5.4	<0.001
Waist circumference (cm)	125.3[a] ± 17.2	114.7[b] ± 16.0	104.1[c] ± 14.5	95.1[d] ± 15.1	<0.001
Fat mass (kg)	47.0[a] ± 11.7	40.7[b] ± 10.8	33.0[c] ± 10.0	27.0[d] ± 10.8	<0.001
Fat-free mass (kg)	62.9[a] ± 8.7	58.5[b] ± 8.4	56.2[c] ± 8.0	54.0[d] ± 7.7	<0.001
Skeletal muscle mass (kg)	59.7[a] ± 8.3	55.6[b] ± 8.0	53.4[c] ± 7.6	51.2[d] ± 7.3	<0.001
Basal metabolic rate (kcal/day)	1960.0[a] ± 275.2	1811.9[b] ± 259.7	1716.6[c] ± 241.1	1632.5[d] ± 229.5	<0.001
**Inflammatory Markers**					
Procalcitonin (ng/mL)	0.030[a] (0.01–0.16)	0.020[b] (0.00–0.06)	0.020[b] (0.01–0.07)	0.030[b] (0.00–0.12)	<0.001
SII Index	40.06[a] (21.15–68.75)	20.01[b] (2.15–68.18)	22.44[b] (2.80–56.25)	19.95[b] (7.76–80.84)	<0.001*
SIRI Index	0.99[a] (0.40–1.68)	0.78[ab] (0.24–2.47)	0.65[b] (0.27–2.14)	0.73[ab] (0.16–3.46)	<0.001*

Parameter	Preoperative	Postoperative Month 1	Postoperative Month 3	Postoperative Month 6	p value
Ferritin (ng/mL)	29.00[a] (0.85–142.00)	52.00[b] (6.10–131.00)	43.90[b] (3.12–210.00)	34.45[ab] (0.44–171.60)	0.013
Iron (µg/dL)	70.23[a] (29–124)	70.23[a] (29–96)	78.79[b] (66–124)	70.23[a] (29–124)	<0.001
**Glucose Metabolism**					
Glucose (mg/dL)	91.00[a] (69–242)	90.5[a] (62–123)	84.77[b] (59–122)	82.00[b] (69–94)	<0.001
HbA1c (IFCC, mmol/mol)	41.00[a] (13.11–116.40)	38.18[b] (31.20–44.30)	35.98[b] (31.20–39.90)	37.70[b] (29.43–45.40)	<0.001
HbA1c (%)	5.90[a] (2.63–12.80)	5.80[b] (4.64–9.00)	5.43[b] (5.00–5.80)	5.60[b] (4.87–6.64)	<0.001
HOMA-IR	4.22[a] (0.78–40.13)	2.78[b] (0.48–13.50)	2.15[b] (0.74–5.06)	1.81[b] (0.48–5.38)	<0.001
Insulin (µIU/mL)	16.80[a] (3.35–147.75)	12.92[ab] (2.08–565.00)	10.21[b] (4.18–21.73)	9.35[b] (2.29–24.47)	<0.001
C-peptide	3.58[a] (1.50–15.46)	3.03[a] (1.19–6.85)	2.83[b] (1.61–5.39)	2.62[b] (1.09–5.34)	<0.001
**Lipid Profile**					
Triglycerides (mg/dL)	137.0[a] (57–517)	109.0[ab] (71–278)	116.0[b] (46.1–239)	102.0[b] (65–254)	<0.001

Parameter	Preoperative	Postoperative Month 1	Postoperative Month 3	Postoperative Month 6	p value
LDL cholesterol (mg/dL)	111.0^{ab} (2–192)	108.0^{b} (62–237)	132.0^{a} (51–205)	116.0^{ab} (77–193)	0.009
HDL cholesterol (mg/dL)	46.63^{a} (32–69)	37.30^{b} (26.9–59)	42.00^{ab} (28–61.2)	50.30^{a} (29.8–53.4)	0.001
VLDL cholesterol (mg/dL)	27.0^{a} (11–103)	21.0^{b} (14–56)	23.0^{ab} (12–48)	22.32^{b} (15–65)	0.002
Non-HDL cholesterol (mg/dL)	144.0^{ab} (42–271)	129.0^{b} (19–275)	150.0^{a} (89–247)	137.0^{ab} (15–244)	0.008
Total cholesterol (mg/dL)	182.0^{a} (95–323)	175.0^{b} (116–334)	201.0^{a} (119–285)	182.0^{ab} (139–291)	0.001
**Liver Enzymes**					
AST (U/L)	20.0^{ab} (11–60)	24.0^{a} (16–72)	21.0^{b} (13–36)	19.0^{b} (11–31)	<0.001
ALT (U/L)	19.5^{a} (10–92)	31.0^{ab} (13–121)	27.89^{ab} (8–57)	19.0^{ab} (2.20–132)	<0.001
GGT (U/L)	27.0^{a} (5–59)	22.5^{a} (0–49)	17.0^{a} (1.71–245)	13.73^{b} (2.31–36.70)	<0.001

Values are presented as mean ± standard deviation for anthropometric parameters and median (minimum–maximum) for biochemical variables. Comparisons among the four time points were performed using repeated-measures analyses. Different superscript letters indicate statistically significant differences between time points in post hoc pairwise comparisons, whereas identical letters indicate no significant difference. Statistical significance was defined as $p < 0.05$. Abbreviations: BMI, body mass index; SII, systemic immune-inflammation index; SIRI, systemic inflammation

response index; HOMA-IR, homeostasis model assessment of insulin resistance; HDL, high-density lipoprotein; LDL, low-density lipoprotein; VLDL, very-low-density lipoprotein; GGT, gamma-glutamyl transferase; AST, aspartate aminotransferase; ALT, alanine aminotransferase.

Figure S2. Longitudinal Changes in PCT Levels in Female Patients

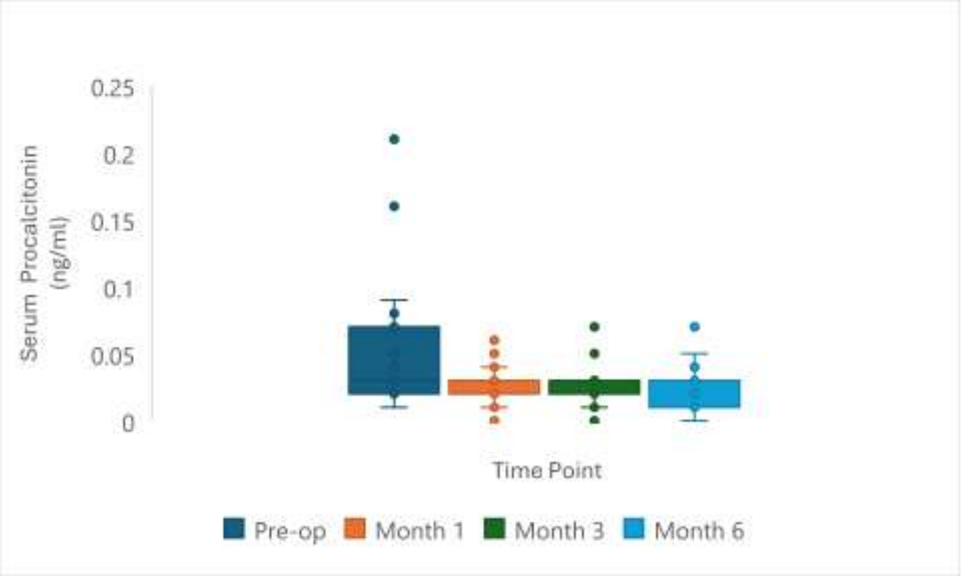

Supplement: Supplementary file 1 [file jcm-15-05293-s001.zip › jcm-4362544-supplementary.pdf]
